# MicroRNAs in Synovial Pathology Associated With Osteoarthritis

**DOI:** 10.3389/fmed.2020.00376

**Published:** 2020-08-11

**Authors:** Ghazaleh Tavallaee, Jason S. Rockel, Starlee Lively, Mohit Kapoor

**Affiliations:** ^1^Arthritis Program, Krembil Research Institute, University Health Network, Toronto, ON, Canada; ^2^Department of Laboratory Medicine and Pathobiology, University of Toronto, Toronto, ON, Canada; ^3^Department of Surgery, University of Toronto, Toronto, ON, Canada

**Keywords:** osteoarthritis, synovium, microRNA, inflammation, fibrosis

## Abstract

Osteoarthritis (OA) is the most common type of arthritis, a disease that affects the entire joint. The relative involvement of each tissue, and their interactions, add to the complexity of OA, hampering our understanding of the underlying molecular mechanisms, and the generation of a disease modifying therapy. The synovium is essential in maintaining joint homeostasis, and pathologies associated with the synovium contribute to joint destruction, pain and stiffness in OA. MicroRNAs (miRNAs) are post-transcriptional regulators dysregulated in OA tissues including the synovium. MiRNAs are important contributors to OA synovial changes that have the potential to improve our understanding of OA and to act as novel therapeutic targets. The purpose of this review is to summarize and integrate current published literature investigating the roles that miRNAs play in OA-related synovial pathologies including inflammation, matrix deposition and cell proliferation.

## Introduction

Osteoarthritis (OA) is the most common chronic debilitating disease imposing a significant socioeconomic burden and affecting the quality of life of millions of people worldwide (1). OA affects the whole joint and involves progressive articular cartilage degradation, subchondral bone remodeling, ectopic bone formation, ligament degeneration, menisci degradation, and synovial inflammation and hypertrophy (2). Many OA studies largely focus on cartilage health as it facilitates joint movement and is highly susceptible to OA; however other tissues, notably the synovium, are now recognized to be involved in OA pathology (3, 4). OA alters the homeostatic functions of cells residing in the synovium, but we are only starting to elucidate the underlying gene expression and regulatory mechanisms responsible, and how these changes contribute to disease progression. Gene expression profiles of the synovium are also altered during OA, which is accomplished by multiple regulatory mechanisms. At the post-transcriptional level, gene transcripts are regulated by a class of small non-coding RNAs called microRNAs (miRNAs). A single miRNA can target a large number of transcripts contributing to tissue specific gene expression (5). The complex network of miRNAs that regulate the pathophysiology of cartilage degeneration during OA has been previously reviewed (6); however, very little is known regarding the role of miRNAs in regulating synovial gene expression during OA. In this review, we summarize the contributions of the synovium to OA pathology and how focusing on the role of miRNAs in regulating the activity of fibroblast-like synoviocytes (FLS) warrants further study to further elucidate mechanisms contributing to OA pathologies.

## Cellular Interactions in the OA Synovium

The synovium is a loose connective tissue that encapsulates the joint and aids in maintaining joint homeostasis through the functions of its resident cells: synovial macrophages and the more abundant FLS [reviewed in (3, 4)]. FLS are mesenchyme-derived cells that share characteristics with other fibroblasts, such as the expression of collagens IV and V, vimentin and CD90, but also show unique expression that differentiates them from other resident fibroblasts, notably cadherin-11 expression by FLS in the synovial lining (7). In healthy synovium, FLS are the major source of extracellular matrix (ECM) and synovial fluid, while resident macrophages remove metabolites and products of matrix degradation (4). As OA progresses, the synovium undergoes hyperplasia, sublining fibrosis, increased vascularization, and increased cell proliferation, migration and invasion (3).

In the context of OA, FLS are the major contributors to the observed excessive synovial ECM deposition and fibrosis (8). While they are involved in the production of proinflammatory and profibrotic mediators, resident synovial macrophages also respond and contribute to OA progression and inflammatory responses (9). Accumulation of macrophages in the synovium is a defining characteristic of synovitis, notably adjacent to areas of cartilage degradation (10, 11). Macrophages are highly plastic cells; and although a broad spectrum of activated states exists, macrophages are generally classified as pro-inflammatory (M1) and inflammatory resolving (M2) (12). In healthy conditions, macrophages are thought to be in an M2-like phenotype that maintains tissue homeostasis and repair (13). Inflammatory mediators, such as interleukin 1β (IL-1β) and tumor necrosis factor α (TNF-α), as well as catabolic enzymes, such as matrix metalloproteases (MMPs) and a disintegrin and metalloproteinase with thrombospondin motifs (ADAMTSs), are produced by synoviocytes and secreted into synovial fluid in quantifiable levels (9). These changes contribute to the excessive ECM deposition and increased synovial thickness detected in OA patients and animal models, and impact joint integrity.

In the synovial fluid of patients with knee OA, the balance of M1 and M2 macrophage markers is skewed toward a pro-inflammatory state, and the degree of the shift is positively associated with the OA severity (14). In the OA synovium, the majority of macrophages possess pro-inflammatory profiles, driving responses that promote synovitis and osteophyte formation (10, 11, 15). In addition to modulating local inflammatory responses, activated macrophages secrete various MMPs and ADAMTSs, which remodel the synovial matrix, and enhance fibrosis-promoting activities of FLS (16). The master driver of fibrosis is transforming growth factor-beta 1 (TGF-β1) as it stimulates FLS expression of other profibrotic mediators, including α-smooth muscle actin (α-SMA), vascular endothelial growth factor (VEGF), procollagen-lysine, 2-oxoglutarate 5-dioxygenase 2 (PLOD2), tissue inhibitors of metalloproteinases-1 (TIMP-1), and collagen type I, as well as OA FLS proliferation and migration (17). FLS in turn influence macrophage activity (18). Thus, interactions of FLS with macrophages can also contribute to the pathological changes in the synovium during OA and is an important consideration for future studies.

## miRNA Biogenesis and Function

MicroRNAs (miRNAs) are single stranded endogenous small non-coding RNA molecules of 21–24-nucleotide (nt) length that are transcribed by RNA polymerase II. MiRNAs are expressed in polyadelynated and capped nascent transcripts ~ 200 nt (pri-miRNA) with hairpin structures. Pri-miRNAs are recognized by DiGeorge syndrome critical region gene 8 (Dgcr8, an RNA binding protein) and cleaved into ~70 nt stem loop precursors (pre-miRNA) in the nucleus by Drosha, a nuclease of the RNase III family, and transported to cytoplasm by Exportin 5. Pre-miRNAs are processed into miRNA duplexes in the cytoplasm by the enzyme Dicer. One strand (mature miRNA) asymmetrically assembles into the Argonaute (AGO) protein of the RNA-induced silencing complex (RISC) and the other one is destroyed. Mature miRNAs then bind mRNAs of target genes in a sequence-specific manner via “seed” sequences, 2–8 nucleotides from the 5′ end of miRNAs, usually resulting in cleavage of target mRNAs or translational repression [reviewed in detail in (19, 20)].

## miRNAs in OA Synovial Pathology

OA studies to date mostly focus on the role of miRNAs in regulating cartilage maintenance and degradation. However, miRNAs also regulate other aspects of OA, including synovial pathology. This is an understudied area and consequently, much less is known. For the purpose of this review, we searched PubMed using “Osteoarthritis + synovium + miRNA” and “Osteoarthritis + synovitis + miRNA” for studies published until March 2020. A total of 83 articles were identified. Thirty-five articles focused exclusively on articular cartilage or tissues other than synovium or on OA symptoms, rather than synovial pathologies, leaving 48 articles relevant to this review.

**Figure 1:**
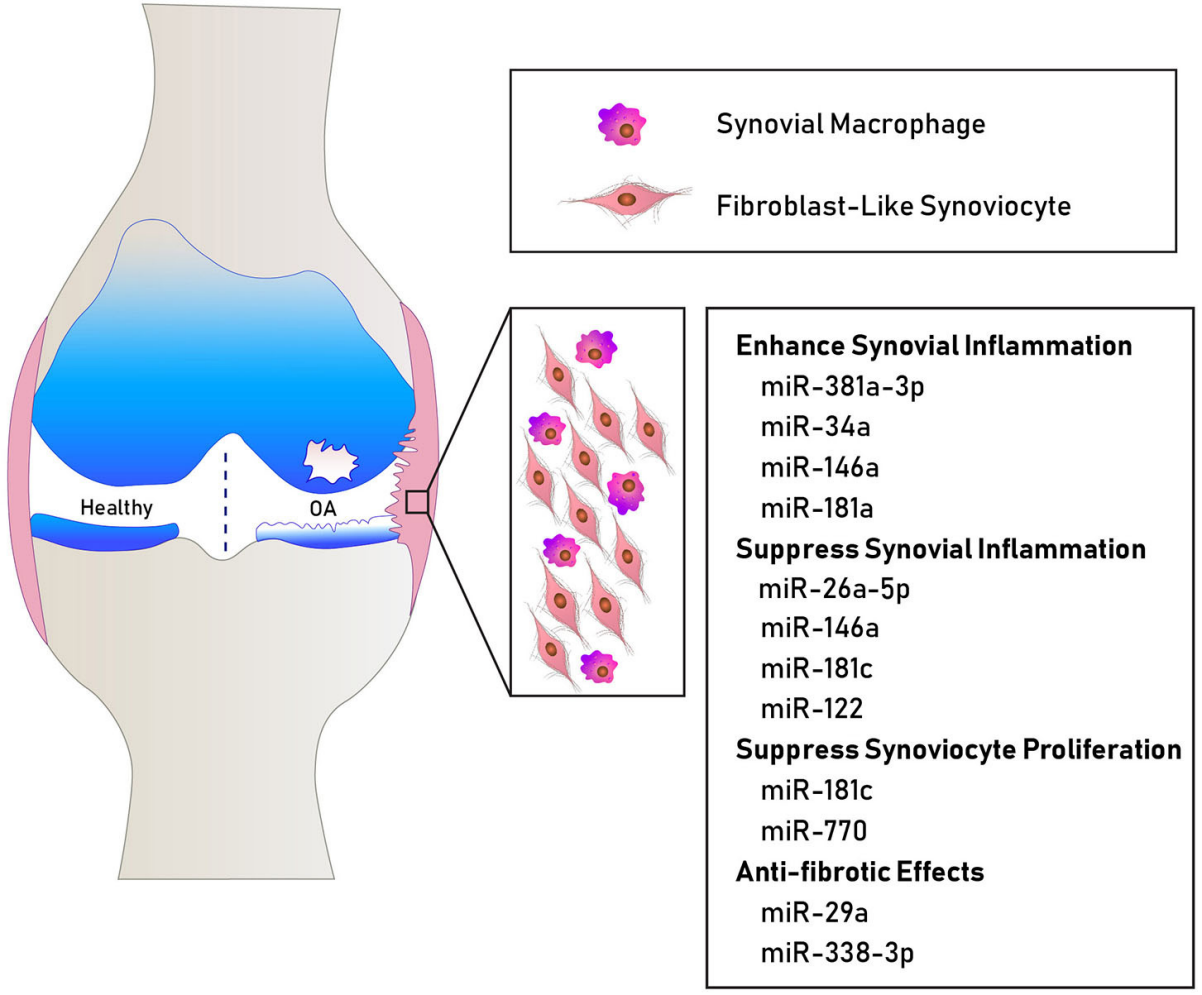
Schematic showing some miRNAs involved in human OA synovial pathology. MiR-381a-3p, miR-34a, miR-146a, and miR-181a promote inflammatory mechanisms (32, 33). MiR-26a-5p, miR-146a, miR-122, and miR-181c suppress the expression of inflammatory cytokines (24, 26, 27, 31). MiR-181c and miR-770 suppress fibroblast-like synoviocyte proliferation (24, 25). MiR-29a and miR-338-3p exhibit anti-fibrotic effects (34, 35).

Considering FLS as essential participants in joint homeostasis and contributors to OA synovial pathology, it is not surprising that OA FLS show differential miRNA profiles. Recently, deep sequencing identified 245 differentially expressed genes in OA FLS and bioinformatics analyses highlighted “ECM organization and altered cellular movement” as one of the most enriched OA FLS functions connected to the differentially expressed genes and miRNA network (21). OA FLS also exhibit an independent miRNA signature from rheumatoid arthritis (RA) FLS, negatively correlating to the expression levels of their putative target genes (22). Elevated levels of miR-625 and miR-124 in OA FLS are associated with decreased expression of their target genes, while miR-155b and miR-203 are expressed at lower levels concomitant with higher expression of their target genes (22). In addition to *in vitro* studies, animal models aid in the understanding of differentially expressed miRNAs in OA synovium. Kung et al. found 394 miRNAs transiently expressed at 1 vs. 6 weeks in the synovium of the destabilization of the medial meniscus (DMM) mouse model of knee OA (23). Thus, several miRNAs modulated in the synovium potentially contribute to joint destruction, synovial inflammation, and fibrosis (summarized in Table 1 and Figure 1). However, the individual and combined contributions of these miRNAs to synovial pathology warrant further investigation to comprehensively understand their role and signaling mechanisms in OA.

**Table 1:** Role of some miRNAs in the synovial pathology during OA.

**MiRNA**	**Species/model system**	**Role in OA**	**References**
miR-181c	Human OA FLS	Suppresses expression of MMP13, IL-6, and IL-8 and targets OPN to reduce FLS proliferation.	(24)
miR-770	Human OA FLS	Suppresses proliferation of OA FLS.	(25)
miR-26a-5p	Human OA FLS	Targets COX2 to reduce Bcl-2, IL-6, TNF-α, and IL-8 expression.	(26)
	Rat instability model of OA	Alleviates synovial inflammation.	
miR-146a	Human OA FLS	Dampens IL-1β signaling.	(27–29)
	Mouse Knockout	Exhibits synovial hyperplasia.	(30)
miR-122	Human OA FLS	Reduces IL-1α levels.	(31)
miR-381a-3p	Human OA FLS	Targets IκBα to enhance NF-κB activity.	(32)
	Rat MIA	Upregulates in the synovium of MIA rats.	
miR-34a miR-146a miR-181a	Human OA FLS	Promote inflammatory mechanisms and oxidative stress.	(33)
miR-29a	Human OA FLS	Targets VEGF and suppresses ECM production.	(34)
	Mouse CIOA	Protects the synovium from hyperplasia and macrophage infiltration.	
miR-338-3p	Human OA FLS	Targets TRAP-1 to regulate TGF-β responsive genes.	(35)
miR-125	Human HUVEC	Enhances glycolysis and angiogenesis.	(36)
miR-128	Mouse ACLT	Promotes synovial membrane thickness and fibroblast activation.	(37)
miR-101	Rats MIA	MiR-101 inhibition reduces cytokine expression in the MIA rats synovium.	(38)

*ACLT, anterior cruciate ligament transection; CIOA, collagenase-induced osteoarthritis; FLS, fibroblast-like synoviocytes; HUVEC, human umbilical vein endothelial cells; OA, osteoarthritis; MIA, monosodium iodoacetate; ECM, extracellular matrix; MMP13, matrix metalloprotease 13; IL-6, interleukin-6; IL-8, interleukin-8; OPN, osteopontin; COX2, cyclooxygenase-2; Bcl-2, B-cell lymphoma-2; TNF-α, tumor necrosis factor alpha; IL-1β, interleukin-1 beta; IL-1α, interleukin-1 alpha; IκBα, inhibitor of nuclear factor kappa B alpha; NF-κB, nuclear factor kappa-light-chain-enhancer of activated B cells; VEGF, vascular endothelial growth factor; TRAP-1, TNF receptor-associated protein-1; TGF-β, transforming growth factor-beta*.

## miRNAs and Synovial Inflammation in OA

MiRNAs play key roles in OA-related synovial inflammation. The expression levels of inflammatory-related miRNAs measured in the synovium from OA patients and animal models show unique signatures when compared to normal controls. When comparing inflamed areas with normal areas of synovium from OA patients, 31 miRNAs are identified in an OA-specific regulatory network comprised of 97 interactions of 38 transcription factors and 35 genes (39). Many miRNAs are upregulated during OA that exacerbate inflammatory responses in the synovium. MiR-381a-3p is upregulated in the synovium of both OA patients and in the monosodium iodoacetate (MIA)-injected rat model of OA pain; and miR-381a-3p enhances nuclear factor kappa-light-chain-enhancer of activated B cells (NF-κB) activity in cultured human OA FLS by targeting inhibitor of nuclear factor kappa B alpha (IκBα) (32). Inhibition of miR-101 in MIA-injected rats reduces cytokine expression in the synovium (38). Furthermore, blocking miR-128 reduces both synovial membrane thickness and fibroblast activation protein (FAP)-positive FLS accumulation in the mouse anterior cruciate ligament transection (ACLT) model of OA (37). Thus, fine-tuning synovial inflammation through miRNA modulation is a promising avenue of research for future OA therapeutic targets.

Some miRNAs have been shown to exhibit anti-inflammatory effects in the synovium during OA. For instance, administration of human bone mesenchymal stem cell-derived exosomes overexpressing miR-26a-5p to cultured OA FLS targets cyclooxygenase-2 (COX2), reducing B-cell lymphoma 2 (Bcl-2), IL-6, TNF-α and IL-8 expression, and increasing Bcl-2-associated X protein (Bax) expression and caspase cleavage, alleviating synovial inflammation in a rat joint instability model of OA (26). MiR-146a is highly expressed in the synovium during OA and when knocked-down in mouse models, NOTCH1 and IL-6 expression are increased in the synovium concomitant with synovial hyperplasia (30). When overexpressed in OA FLS, miR-146a decreases the expression of inflammatory mediators, including IL-1-induced TNF receptor associated factor 6 (TRAF 6), IL-1 receptor-associated kinase 1 (IRAK 1), COX2, IL-8, MMP13, and ADAMTS5 expression (27). Denbinobin, a naturally occurring 1,4-phenanthrenequinone, promotes histone acetyltransferase activity, resulting in increased miR-146a expression and inhibition of nuclear factor (NF)-κB activity, dampening IL-1β-elicited expression of cell adhesion molecules and monocyte adhesion to OA FLS (28). Intriguingly, histone deacetylase inhibitors also promote miR-146a expression in IL-1β-treated OA FLS by facilitating NF-κB binding to miR-146a promoter, which reduces downstream responses including IL-6 secretion (29). Thus, the acetylation pattern of miR-146a is an important aspect to its expression and function in OA FLS. MiR-122 is another miRNA with anti-inflammatory potential as its overexpression in OA FLS reduces IL-1α levels (31). Taken together, miRNAs have the potential to regulate inflammation positively or negatively in the OA synovium; but timing, source, and context of their expression in relation to OA-related inflammatory responses needs to be better understood.

RA FLS have been shown to mount greater inflammatory responses compared to OA FLS, expressing higher levels of certain inflammation-inducing miRNAs. For instance, miR-146a, miR-155, and miR-223 are expressed at higher levels in synovial tissues and RNA extracted from paraffin embedded RA synovial sections relative to OA samples (40, 41). OA tissue is routinely used as control comparisons in these instances. As a result, much more is known about the role of miRNA in RA FLS and synovial tissue. In RA FLS, miR-155 suppresses MMP1 and MMP3 expression (42). Inhibition of miR-155 in synovial fluid-derived macrophages reduces TNF-α production *in vitro* (43). Mir-221-3p is also expressed at higher levels in RA synovial tissue and fluid, and inhibits the anti-inflammatory arm of macrophages by suppressing the JAK3/STAT3 axis and increasing the expression of inflammatory mediators such as IL-6 and IL-8 (44). Similarly, miR-145-5p and miR-143-3p are expressed at higher levels in RA synovium and FLS compared to OA (45). MiR-145-5p targets osteoprotegerin, aggravating bone erosion in collagen-induced arthritis, and also regulates semaphorin 3A (SEMA3A) to modulate the phenotype of RA FLS (45, 46). MiR-143-3p targets insulin-like growth factor1 receptor (IGF1R) and insulin-like growth factor binding protein 5 (IGFBP5) expression, regulating the Ras/p38 MAPK signaling pathway, contributing to FLS proliferation and apoptosis (45, 47). Additionally, miR-203 promotes NF-κB activation and secretion of MMP1 and IL-6, thereby accelerating RA FLS activation (48). Overall, miRNAs clearly modulate the inflammatory profile of synovial macrophages and FLS in RA.

However, it is now appreciated that OA FLS exhibit an independent miRNA signature from RA FLS (22). Intriguingly, several miRNAs that negatively regulate inflammation or FLS proliferation are expressed at higher levels in OA synovium and FLS compared to RA, including miR-34a-3p, miR-124a, miR-30a, miR-10a, miR-140-3p, and miR-140-5p (49–53). MiR-34a-3p expression is decreased in RA FLS, leading to increased inflammation and proliferation (49). Downregulation of miR-34a passenger strand (miR-34a^*^) in RA FLS, due to methylation of its promoter, promotes apoptosis resistance (54). MiR-124a also suppresses proliferation and inflammation by directly targeting cyclin-dependent kinase 2 (CDK-2) and monocyte chemoattractant protein-1 (MCP-1) in RA FLS (50). Furthermore, decreased levels of miR-30a in RA synovium correlate with reduced apoptosis and enhanced autophagy (51), while lower expression of miR-10a is thought to promote excessive secretion of inflammatory cytokines via NF-κB regulation (52). OA is considered a low-grade inflammatory disease compared to RA or other types of inflammatory arthritis (55); thus, it is not surprising that many miRNAs are differentially expressed in RA compared to OA synovial cells. However, it does not preclude the possibility that these miRNAs also contribute to synovial inflammation and OA progression. Detailed comparisons of the differential miRNA profiles detected in RA and OA synovial cells coupled with mechanistic studies could offer a jumping point for future investigations into their contributions to OA pathogenesis.

## miRNAs and Synovial Fibrosis

In general, a limited number of studies have investigated the role of miRNAs in processes associated with OA synovial fibrosis. For instance, miR-29a targets VEGF and its inhibition in OA FLS promotes the expression of ECM genes (collagen III, TGF-β1, PLOD2, TIMP1, ADAM12, MMP9, MMP13, and ADAMTS5) (34). Conversely, miR-29a overexpression decreases VEGF and ECM gene expression levels. In a mouse model of collagenase-induced OA (CIOA), intra-articular administration of a miR-29a precursor protects the synovium from hyperplasia and macrophage infiltration (34). Thus, miR-29a, which is decreased in OA synovium, appears to reduce profibrotic activities in the healthy synovium by tightly regulating angiogenesis and ECM production. MiR-338-3p is another ECM-regulating miRNA decreased in OA synovium and synovial effusions compared to synovial tissues from patients with joint trauma. MiR-338-3p counteracts TGF-β1-induced expression of vimentin, type I collagen and TIMP1 in FLS by directly targeting TNF receptor-associated protein 1 (TRAP-1) and regulating Smad 2/3 signaling pathways (35). Overall, these miRNAs exhibit anti-fibrotic regulatory effects; however, there are likely more miRNAs with similar activities that remain to be identified in addition to miRNAs with profibrotic effects that exacerbate synovitis associated with OA.

Profibrotic mediators have also been shown to regulate miRNA expression, contributing to OA synovial pathology. For instance, TGF-β1 enhances the expression of anti-inflammatory factor hemeoxygenase 1 (HO-1) by reducing the expression of miRNA-519b in human OA FLS (56). TGF-β1 also inhibits miR-92a to promote the expression of forkhead box class O 3 (FOXO3) in OA FLS, lowering mRNA and protein levels of TNF-α, IL-1β, VEGF, and C-C Motif Chemokine Ligand 2 (CCL2) (57). Another profibrotic growth factor, connective tissue growth factor (CTGF), increases miR-210 expression in OA FLS by activation of PI3K, AKT, ERK, and NF-κB/ELK1 pathways, contributing to VEGF-dependent angiogenesis (58). It is noteworthy that profibrotic mediators such as TGF-β1 and CTGF modulate select miRNAs to regulate certain aspects of synovitis including inflammation and angiogenesis. This effect can be counteracted by other miRNAs. MiR-125a is expressed at higher levels in OA synovium compared to psoriatic arthritis and modulates glycolysis in human umbilical vein endothelial cells (HUVEC) to inhibit angiogenesis (36). MiRNAs are dysregulated in the synovium during OA, but the way in which they regulate inflammation, angiogenesis or ECM modulation, and how they interact to maintain the joint homeostasis, remains poorly understood and requires extensive investigation in near future.

## Mechanisms Regulating miRNAs in OA Synovium

Adipocyte-derived molecules (adipokines) are elevated in the joint during OA and play an important role in cartilage and bone turnover (59). In addition, adipokines alter miRNA expression levels, modulating synovial inflammatory responses. Visfatin and resistin upregulate miR-34a, miR-146a and miR-181a in OA FLS, which when inhibited, decreases proinflammatory responses and oxidative stress (33). Adipokines can also inhibit miRNAs to enhance inflammatory responses. For instance, visfatin inhibits miR-199a-5p expression in OA FLS through ERK, p38, and JNK signaling pathways, which promotes IL-6 and TNF-α production (60). Similarly, resistin suppresses miR-33a and miR-33b in OA FLS resulting in increased MCP-1 transcription, facilitating the migration of monocytes (61). Thus, select miRNAs are regulated by adipokines influencing OA-related inflammatory responses.

Just as miRNAs regulate mRNAs, miRNAs are also regulated through interaction with RNA partners, specifically long non-coding (lnc) RNAs and circular (circ) RNAs (24, 25, 62). Both act as sponges, binding directly to miRNAs and regulating their free concentration. Evidence suggests that these regulatory RNAs have the capacity to fine-tune miRNA activity in OA FLS. For example, lncRNA nuclear enriched abundant transcript 1 (NEAT1) binds miR-181c, inhibiting osteopontin (OPN) expression and regulating OA FLS proliferation (24). Similarly, the lncRNA prostate cancer gene expression marker 1 (PCGEM1) binds miR-770, promoting OA FLS proliferation and survival (25). In fact, 122 circRNAs are differentially expressed in the OA synovium, with over 1,000 miRNAs and 28,000 circRNA-miRNA interaction pairs. Intriguingly, 641 miRNAs are predicted to interact with six circRNAs (62). These findings indicate that many miRNAs can be modulated by a handful of circRNAs, adding complexity to the network that regulates synoviocyte expression profiles. CircRNAs and lncRNAs represent an opportunity to modulate several miRNAs simultaneously, and thus hold great therapeutic potential to modulate OA synovial pathology.

## Future Directions

An important aspect overlooked in many OA studies using animal models is that OA is an age-related disease and experiments are routinely conducted in young animals. As with other organ systems, cellular activity in joint tissues is altered with age, including abnormal ECM, cytokine and reactive oxygen species (ROS) production, which likely contribute to OA pathology differently than post-traumatic or metabolic-induced OA (63, 64). Little is known regarding how aging alters synovial homeostasis and function over time, and how that might contribute to OA progression. Expression of many miRNAs change with age in various tissues, altering processes like cell senescence (65). MiR-126-3p, which is important for cell attachment to the ECM, is downregulated in aged OA chondrocytes relative to their younger counterparts (66). Improving our understanding of how miRNAs are differentially expressed with age, and how this alters joint homeostasis and OA progression will be essential for future translational success.

Integrated analyses examining miRNA and transcript profiles in parallel will help elucidate dysregulated miRNA and RNA interactions occurring in OA. In OA, Chen et al. performed RNA sequencing alongside small RNA sequencing in OA FLS compared to those derived from healthy tissue (21). Putative targets of dysregulated miRNAs were predicted by bioinformatic approaches, including 14 genes (11 upregulated and 3 downregulated) that require further biological validations (21). Another study attempted to identify differential mRNA and miRNA expression in the DMM mouse OA model using microarray and RT-qPCR, but found no evidence of differential expression of miRNAs and RNAs levels between sham and DMM-induced OA mice at 1 or 6 weeks after surgery (23). However, the time after surgery examined, the small sample size used and variability observed within the groups, might be masking some relevant changes, and further investigation is warranted.

In addition to holding therapeutic potential, miRNAs in the synovial fluid or synovium-derived extracellular vesicles (EVs) might also act as biomarkers (67, 68). Increased levels of miR-23a-3p, miR-24-3p, miR-186-5p, miR-29c-3p, miR-34a-5p, and miR-27b-3p are found in the synovial fluid of OA patients with late-stage compared to early-stage radiographic knee OA (69). Some of these miRNAs are highly expressed in the OA synovium. MiR-210 is increased in the synovial fluid of both early- and late-stage radiographic knee OA patients compared to healthy donors and positively correlates with VEGF levels (70). Other synovial fluid miRNAs suggested as OA biomarkers include miR-29b-3p and miR-140, which show positive and negative correlations with radiographic knee OA severity, respectively (71, 72). As we continue to unearth the biomarker potential of some of these miRNAs, understanding the release mechanism as well as the exact cellular source of secreted miRNAs in the joint will advance our understanding of miRNA contributions to OA pathology. Profiling miRNA content of cells and tissues using next generation sequencing not only helps to identify the source of miRNAs, but also has the added advantage of identifying novel miRNAs, expanding the rapidly-growing human miRNA repository and promoting investigations into new regulatory mechanisms and therapeutic targets. Sequencing datasets are routinely deposited on-line, and this open format is not only idea-generating but can also be used to substantiate novel findings. MiRNAs are currently being explored as potential therapeutic targets to counteract cartilage degeneration and synovitis in OA. For example, inhibition of miR-101 and miR-128 has been shown to rescue cartilage degeneration and synovitis in MIA and ACLT animal models of OA, respectively (37, 38). Extensive research is underway to identify the best mode of delivery of miRNA-based therapies (mimics or inhibitors) in preclinical models of OA.

## Conclusions

Taken together, miRNAs contribute to synovial homeostasis, inflammation, fibrosis, angiogenesis, cell survival and cell apoptosis, contributing to OA synovial pathology. MiRNAs have been a focus of OA research since their discovery and they are attracting more attention due to their biomarker and therapeutic potential. However, research on the role of miRNAs in OA-related synovial pathology is only in its infancy. Most research on synovitis is performed in samples from RA patients or animal models where OA tissues are often used as a control reference. This has hampered our understanding of the mechanisms modulated by miRNAs in OA synovitis. Additional studies are needed to comprehensively understand the role miRNAs play in OA-related synovial pathology and to identify novel disease modifying targets for therapeutic development.

## Author Contributions

GT and SL performed the relevant literature searches. GT, JR, and SL wrote the manuscript. MK critically reviewed the manuscript and provided important intellectual and funding contributions. All authors have critically read and approved the manuscript.

## Conflict of Interest

The authors declare that the research was conducted in the absence of any commercial or financial relationships that could be construed as a potential conflict of interest.
